# Intrapartum maternal glycaemic control for the prevention of neonatal hypoglycaemia: a systematic review and meta-analysis

**DOI:** 10.1186/s12884-024-06615-8

**Published:** 2024-06-13

**Authors:** Caitlyn M. Ulyatt, Lily F. Roberts, Caroline A. Crowther, Jane E. Harding, Luling Lin

**Affiliations:** Liggins Institute, University of Auckland, 85 Park Road, Grafton, Auckland, 1023 New Zealand

**Keywords:** Intrapartum, Labour, Glycaemic control, Hypoglycaemia, Neonatology, Infant, Newborn

## Abstract

**Background:**

Neonatal hypoglycaemia is the most common metabolic disorder in infants, and may be influenced by maternal glycaemic control. This systematic review evaluated the effect of intrapartum maternal glycaemic control on neonatal hypoglycaemia.

**Methods:**

We included randomised controlled trials (RCTs), quasi-RCTs, non-randomised studies of interventions, and cohort or case-control studies that examined interventions affecting intrapartum maternal glycaemic control compared to no or less stringent control. We searched four databases and three trial registries to November 2023. Quality assessments used Cochrane Risk of Bias 1 or the Effective Public Health Practice Project Quality Assessment Tool. Certainty of evidence was assessed using the Grading of Recommendations, Assessment, Development and Evaluation (GRADE). Meta-analysis was performed using random-effects models analysed separately for women with or without diabetes. The review was registered prospectively on PROSPERO (CRD42022364876).

**Results:**

We included 46 studies of women with diabetes and five studies of women without diabetes: one RCT, 32 cohort and 18 case-control studies (11,273 participants). For women with diabetes, the RCT showed little to no difference in the incidence of neonatal hypoglycaemia between tight versus less tight intrapartum glycaemic control groups (76 infants, RR 1.00 (0.45, 2.24), *p* = 1.00, low certainty evidence). However, 11 cohort studies showed tight intrapartum glycaemic control may reduce neonatal hypoglycaemia (6,152 infants, OR 0.44 (0.31, 0.63), *p* < 0.00001, I^2^ = 58%, very low certainty evidence). For women without diabetes, there was insufficient evidence to determine the effect of tight intrapartum glycaemic control on neonatal hypoglycaemia.

**Conclusions:**

Very uncertain evidence suggests that tight intrapartum glycaemic control may reduce neonatal hypoglycaemia in infants of women with diabetes. High-quality RCTs are required.

**Supplementary Information:**

The online version contains supplementary material available at 10.1186/s12884-024-06615-8.

## Background

Neonatal hypoglycaemia is the most common metabolic disorder in infants, with an incidence of 5–15% of all births [1–3]. At-risk populations include infants with large or small birthweights, born preterm, or to a mother with diabetes [2]. These at-risk infants have an incidence of hypoglycaemia of 50% [4]. As glucose is the primary oxidative substrate for brain metabolism, low glucose concentrations can lead to seizures, neurodevelopmental impairment and brain injury [2, 5].

Currently, the National Institute for Health and Care Excellence (NICE) guidelines recommend that intravenous dextrose and an insulin infusion should be commenced at the start of labour for women with type 1 diabetes, and for women with other types of diabetes if the blood glucose concentrations are not between 4.0 and 7.0mmol/L [6]. This glycaemic range is also recommended in other national and international clinical practice guidelines [7–10]. The NICE guideline recommendation was based on evidence from 8 observational studies published from 1985 to 2002, which found that babies of mothers with higher intrapartum blood glucose concentrations were at increased risk of neonatal hypoglycaemia [11–18]. The Joint British Diabetes Society for Inpatient Care has recommended target intrapartum glucose concentrations of 4.0-7.0mmol/L in women with diabetes receiving antenatal corticosteroids but, more recently, a more pragmatic target of 5.0-8.0mmol/L [19]. For women without diabetes, glucose concentrations usually remain stable during labour and insulin concentrations are depressed, although interventions during this time may alter glycaemia [20].

The evidence supporting tight intrapartum glycaemic control to reduce neonatal hypoglycaemia has been questioned [21–23]. A systematic review published in 2018 found 6/23 studies reported a significant relationship between intrapartum glucose concentrations and neonatal hypoglycaemia whilst 12/23 found no significant relationship [22]. Since then, several additional studies have been published.

Since intrapartum glycaemic control usually involves insulin plus dextrose infusion coupled with regular monitoring of blood glucose concentrations, tight glycaemic control requires additional resources. Tight control may also increase the risk of adverse effects, including maternal hypoglycemia. Thus, it is crucial to determine the benefits and risks of this intervention. We undertook this systematic review to clarify the effect of intrapartum glycaemic control on neonatal hypoglycaemia among women with and without diabetes [24].

## Methods

We conducted this review following the methodology outlined in the Cochrane Handbook for Systematic Reviews of Interventions [25]. We prospectively registered this review protocol in Prospero (registration number CRD42022364876), and reported using the preferred reporting items for systematic reviews and meta-analyses (PRISMA) guidelines [26] [Supplementary Table 1, Additional file 1].

### Search strategy and selection criteria

We searched MEDLINE (Ovid), Embase (Ovid), CINAHL Complete, and the Cochrane Central Register of Controlled Trials (CENTRAL) from inception to November 30th, 2023. We also searched the Australian and New Zealand Clinical Trials Registry (https://www.anzctr.org.au/), Clinical Trials (www.ClinicalTrials.gov) and the World Health Organisation (WHO) International Clinical Trials Registry Platform (ICTRP) Search Portal (https://apps.who.int/trialsearch/) to identify registered trials. Abstracts from conferences were included if they contained usable data. Reference lists of included studies were also screened [Additional File 2].

Inclusion criteria were published and unpublished randomised controlled trials (RCTs), quasi-RCTs, cluster randomised trials, non-randomised studies of interventions, cohort, or case-control studies about pregnant women and their infants where the intervention was any intervention that changed intrapartum maternal glycaemia (investigator-defined), and the comparator was no intervention or interventions that resulted in less tight control of intrapartum maternal glycaemia. There were no restrictions on language or publication date.

The primary outcomes were neonatal hypoglycaemia and maternal hypoglycaemia (both investigator-defined). Secondary neonatal outcomes were hypoglycaemia (any blood glucose concentration < 2.6 mmol/L) during the initial hospital stay, severe hypoglycaemia (any blood glucose concentration < 2.0 mmol/L or investigator-defined), receipt of treatment for hypoglycaemia during initial hospital stay (investigator-defined, any treatment including oral dextrose gel, intravenous dextrose, or other drug therapy), number of episodes of hypoglycaemia (investigator‐defined), hypoglycaemic injury on brain imaging, admission to special care nursery or neonatal intensive care nursery, admission to special care nursery or neonatal intensive care nursery for hypoglycaemia, stillbirth/neonatal death, Apgar score < 7 at 5 min, respiratory distress syndrome (investigator-defined), breastmilk feeding exclusively (infant only receives breast milk without any other drink or food) from birth to discharge, duration of initial hospital stay, adverse effects (investigator-defined), and developmental impairment at follow-up (investigator-defined). Secondary maternal outcomes were glycaemic control achieved (compliance with the glycaemic targets being used, investigator-defined), use of additional intrapartum pharmacological treatment for maternal glycaemic control, adverse effects of the intervention (investigator-defined), duration of labour, mode of birth (vaginal or caesarean birth), postpartum haemorrhage (investigator-defined), postpartum infection (investigator-defined), postnatal depression (investigator-defined), diabetic ketoacidosis, satisfaction with intrapartum treatment/care, costs associated with the intervention (investigator-defined), cost of maternal care, and cost of offspring care.

### Data collection and analysis

Two reviewers (CU and LR) used Covidence [27] to independently screen the titles and abstracts of identified records, determine eligibility for inclusion of full-text articles and extract the data onto a pre-specified data extraction form. Study outcomes, setting, inclusion and exclusion criteria, authors’ declaration of interest, funding sources, ethics approval, baseline characteristics and intervention and comparison details were all recorded. Articles not published in English were translated by a colleague or DeepL [28]. The risk of bias was assessed by two independent reviewers (CU and LR) using the Cochrane Risk of Bias 1 tool (RoB1) [29] for RCTs and quasi-RCTs and the Effective Public Health Practice Project (EPHPP) Quality Assessment Tool for Quantitative Studies [30] for non-randomised studies, cohort studies, and case-control studies. Disagreements were resolved through discussion between the two reviewers or with a third reviewer (LL).

The certainty of evidence for each key outcome was assessed using the Grading of Recommendations Assessment, Development and Evaluation (GRADE) approach [31] and the GRADEpro Guideline Development Tool (GDT) [32] was used to generate a summary of findings table. Neonatal outcomes considered for GRADE assessment were neonatal hypoglycaemia (investigator-defined), receipt of treatment for hypoglycaemia during initial hospital stay, hypoglycaemic injury on brain imaging, special care nursery or neonatal intensive care nursery admission for hypoglycaemia, breastmilk feeding exclusively from birth to discharge, duration of initial hospital stay, and developmental impairment at follow-up. Maternal outcomes included for GRADE assessment were maternal hypoglycaemia, glycaemic control achieved, use of additional intrapartum pharmacological treatment for maternal glycaemic control, adverse effects of the intervention, mode of birth, satisfaction with intrapartum treatment/care, and costs associated with the intervention.

### Statistical analysis

Meta-analysis was performed using RevMan 5.4.1 [33] or R [28]. We used random-effect models and calculated odds ratios (ORs) and relative risks (RRs) with 95% confidence intervals (CIs) for dichotomous outcomes, and mean differences (MDs) with 95% CIs for continuous outcomes. R software was utilised to pool adjusted ORs for cohort and case-control studies. Statistical significance for all models was denoted as *p* < 0.05. For studies that provided a lower quartile, median and upper quartile or a minimum, maximum and median, the mean and standard deviation were estimated to include the data in the meta-analysis [34]. *χ*^2^ and *I*^2^ were calculated to identify statistical heterogeneity. Significant heterogeneity was an *I*^2^ > 50% and *p* < 0.10, in which case we explored possible causes in sensitivity analyses. For outcomes with more than 10 trials, publication bias was assessed using funnel plots. We planned to conduct subgroup analyses to see if the effect of intrapartum maternal glycaemic control differed for women with different types of diabetes, infants born preterm versus at term, infants at risk of hypoglycaemia versus not at risk, and single versus multiple births. We constructed a characteristic of studies table to determine which studies were suitable for each synthesis [Supplementary Table 2, Additional File 3]. Unless stated, all analyses were prespecified.

## Results

**Fig. 1:**
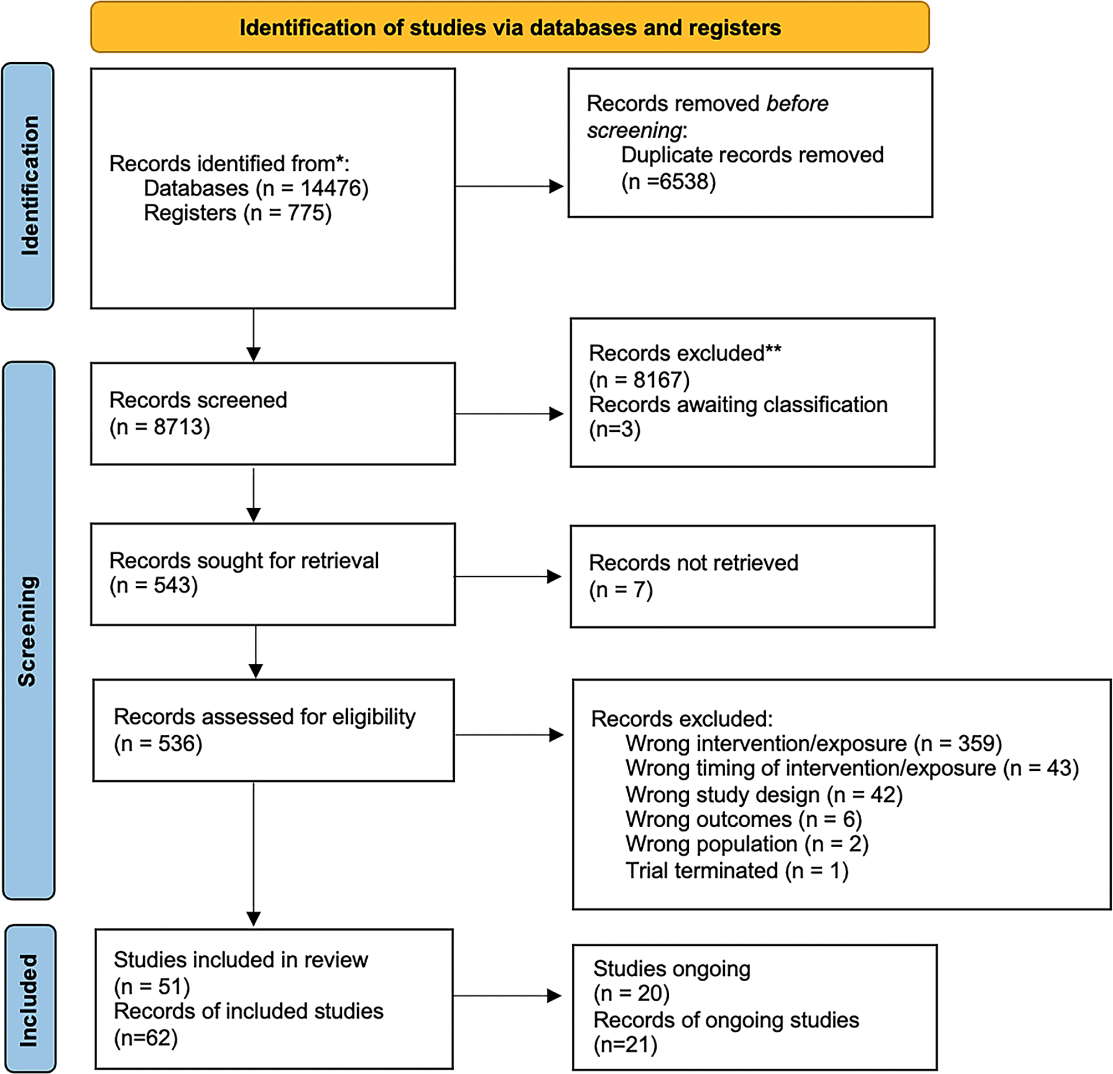
PRISMA flow diagram of included studies

Searching identified a total of 15,251 records, of which 8,713 were title and abstract screened following the removal of duplicates, and 536 full text records screened. Seven records could not be retrieved. Fifty-one studies from 62 records met inclusion criteria and were included in this review. Additionally, there were 21 records of 20 ongoing studies (Fig. 1).

Among the 51 included studies, one was an RCT, eight were prospective cohort studies, 10 were retrospective cohort studies, 18 were case-control studies, and 14 could not be included in the meta-analysis due to lack of a comparator group or raw data [Supplementary Table 2, Additional File 3]. The studies were conducted between 1973 and 2023. According to the 2022 World Bank classification [35], the single RCT included was conducted in the US, a high-income country. In the remaining study designs, 45 studies were conducted in high-income countries, two in upper-middle-income countries, two in lower-middle-income countries, and one in a low-income country. Sample sizes ranged from 16 to 3,680 infants.

### Risk of bias or quality of included studies

**Fig. 2:**
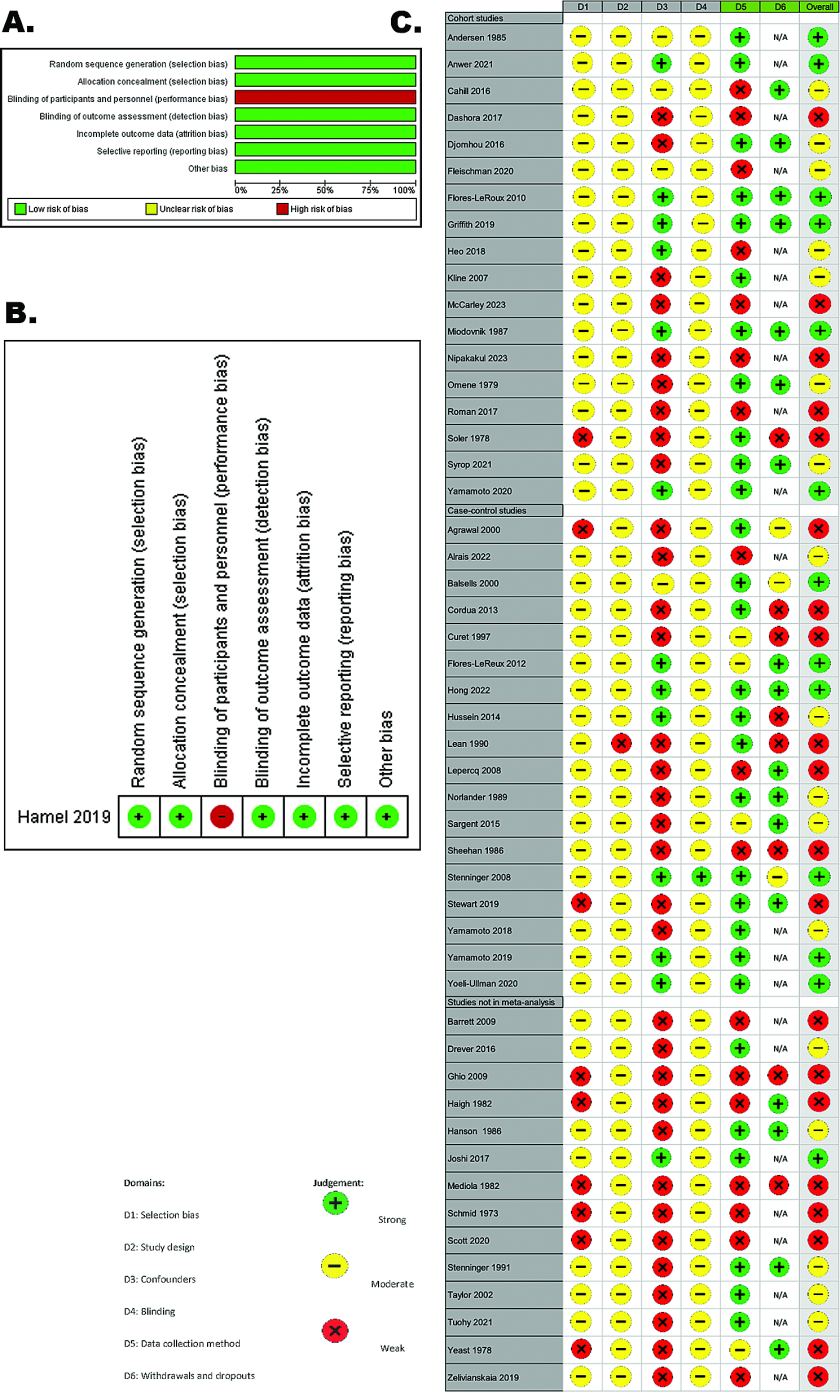
Risk of bias assessment, summary and quality assessment. (**A**) Cochrane Risk of Bias tool 1 risk of bias graph: each domain is represented as a percentage for the single RCT. (**B**) Cochrane Risk of Bias tool 1 risk of bias summary graph for the single RCT included. (**C**) Effective Public Health Practice Project quality assessment graph for the 50 observational studies

The single RCT was at high risk for performance bias (no blinding of participants or personnel) and low risk for all other domains (Fig. 2). Of the 50 remaining studies, 13 were of strong quality overall, 17 were of moderate quality, primarily due to weak methodology in confounder adjustment, and 20 were of weak quality due to weak methodology in two or more domains, largely confounder adjustment and data collection methods.

### A: Women with diabetes

#### Primary outcome: neonatal hypoglycaemia

**Fig. 3:**
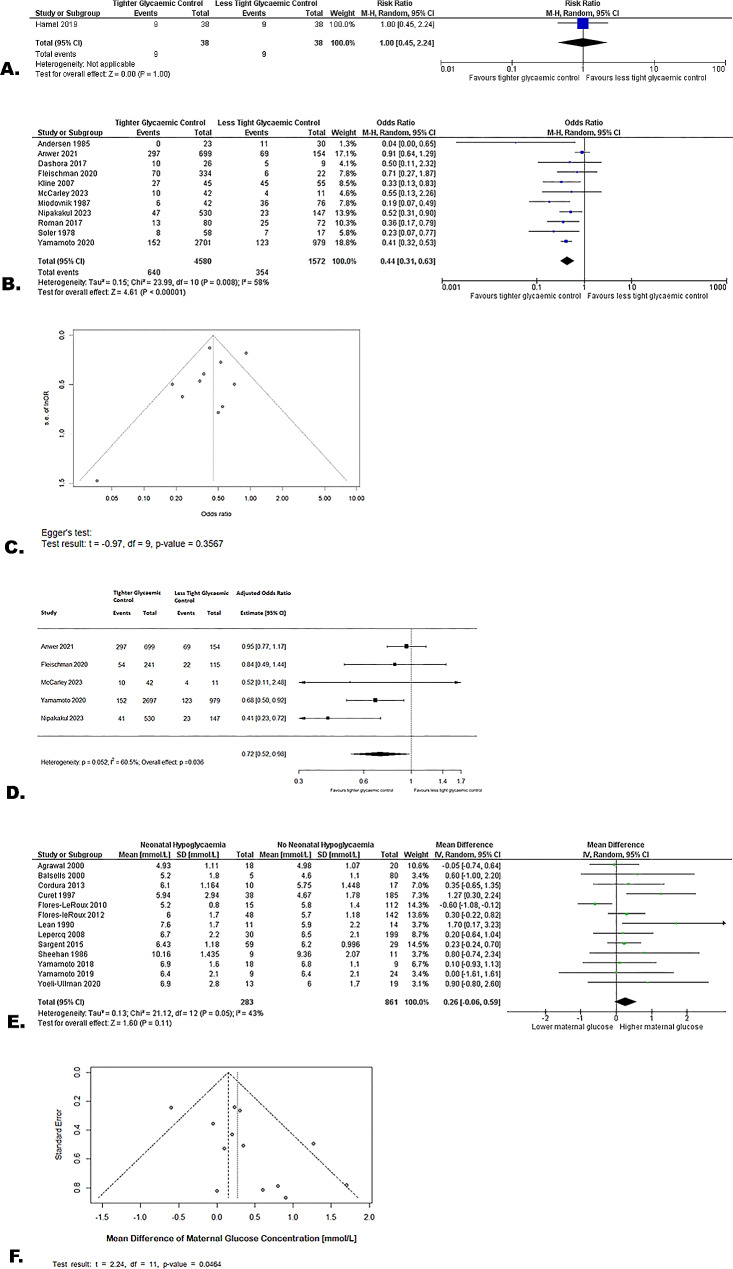
Effect of tight compared to less tight or no intrapartum glycaemic control in women with diabetes on neonatal hypoglycaemia. (**A**) Results from one randomised controlled trial (**B**) Results from 11 cohort studies (**C**) Funnel plot for 11 cohort studies (**D**) Results from the forest plot for cohort studies reporting adjusted values (**E**) Results from 13 case control studies (**F**) Funnel plot for 13 case-control studies

In the RCT, the target blood glucose concentration range was 3.9-5.6mmol/L in the tight intrapartum glycaemic control group, with point-of-care blood testing every hour, and short-acting insulin was initiated if glucose concentrations were > 5.6mmol/L [21]. In the less tight control group, the target range of blood glucose concentration was 3.9-6.7mmol/L, with point-of-care testing every four hours and short-acting insulin was initiated if glucose concentrations were > 6.7mmol/L. Evidence from this RCT showed that tight intrapartum glycaemic control compared with less tight control results in little to no difference in neonatal hypoglycaemia (76 infants, RR 1.00 (0.45, 2.24), *p* = 1.00, low certainty evidence, Fig. 3a).

Evidence from 11 cohort studies showed that tight glycaemic control may be associated with reduced neonatal hypoglycaemia, but the evidence is very uncertain (6,152 infants, OR 0.44 (0.31, 0.63), *p* < 0.0001, I^2^ = 58%, very low certainty evidence, Fig. 3b). Exclusion of Anwer 2021 [36], which showed no difference between groups, from the meta-analysis led to an I^2^ of 0%, suggesting that this study was an important contributor to heterogeneity. The funnel plot did not suggest bias due to small sample sizes (p for Egger’s test = 0.36, Fig. 3c). Evidence from five cohort studies reporting adjusted values were consistent in direction with the unadjusted values (5,615 infants, aOR 0.72 (0.52, 0.98), *p* = 0.04, I^2^ = 60.5%, Fig. 3d).

Evidence from 13 case-control studies showed little to no difference in maternal blood glucose concentrations between those with neonatal hypoglycaemia and those without, but the evidence is very uncertain (1,144 infants, MD 0.26 (-0.06, 0.59), *p* = 0.11, I^2^ = 43%, Fig. 3e). Exclusion of Flores-Le Roux 2010 [37], which was the only study reporting increased neonatal hypoglycaemia with lower maternal glucose concentrations, reduced the I^2^ value to 0%. The funnel plot did suggest bias due to small sample sizes (p for Egger’s test = 0.05, Fig. 3f).

Twelve studies were not included in the meta-analysis as they did not contain any usable data (missing raw data or a comparison group). Seven studies [18, 23, 38–42] found no relationship between maternal intrapartum glucose concentrations and neonatal hypoglycaemia. Five studies [43–48] found a positive relationship between maternal intrapartum glucose concentrations and neonatal hypoglycaemia. No studies found a negative association between maternal glucose concentrations and neonatal hypoglycaemia.

#### Primary outcome: Maternal hypoglycaemia

Evidence from the RCT showed that tight intrapartum glycaemic control may increase maternal hypoglycaemia (76 infants, RR 2.00 (0.54, 7.42), *p* = 0.30, low certainty evidence). Evidence from one cohort study is very uncertain about the association between tight glycaemic control and maternal hypoglycaemia (52 infants, OR 0.13 (0.01, 2.56), *p* = 0.18, very low certainty evidence). One case-control study found no cases of maternal hypoglycaemia in mothers of infants with and without neonatal hypoglycaemia [49].

### Secondary outcomes

#### Neonatal

The RCT reported that tight intrapartum glycaemic control has little to no effect on the receipt of treatment for hypoglycaemia, whereas evidence from observational studies suggest that tight glycaemic control may be associated with a reduction in the receipt of treatment for hypoglycaemia [Additional File 4, Figure S2]. Evidence from the RCT found tight intrapartum glycaemic control has little to no effect on breastmilk feeding exclusively from birth to discharge. Evidence from several observational studies found tight intrapartum glycaemic control may be associated with a reduction in severe hypoglycaemia [Additional File 4, Figure S1], admission to special care or neonatal intensive care nursery [Additional File 4, Figure S3], admission for hypoglycaemia, stillbirth/neonatal death, Apgar score < 7 at 5 min, respiratory distress syndrome, duration of initial hospital stay, and adverse effects, and an increase in developmental impairment at follow-up, but the evidence is very uncertain for all these outcomes (Table 1).

**Table 1:** Effect of tight versus looser intrapartum glycaemic control in diabetic women on secondary outcomes

Outcome	Types and numbers of studies	Number of participants	RR/OR/MD (95% CI)	*P*-value for overall effect	I^2^ for heterogeneity
**Neonatal Outcomes**					
Severity of hypoglycaemia	Cohort: 3	246	OR 0.18 (0.09, 0.38)^a^	<0.0001	0%
	Case-control: 6	555	MD 0.13 (-0.18, 0.45) mmol/L^b^	0.40	0%
Receipt of treatment for hypoglycaemia during initial hospital stay	RCT: 1	76	RR 1.13 (0.67, 1.92) ^a^	0.64	-
	Cohort: 3	3,786	OR 0.38 (0.18, 0.80) ^a^	0.01	32%
	Case-control: 4	89	MD 0.92 (0.19, 1.65)mmol/L^b^	0.01	25%
Admission to special care nursery or neonatal intensive care nursery	RCT: 1	76	RR 5.00 (0.61, 40.81) ^a^	0.13	-
	Cohort: 4	1,077	OR 0.45 (0.28, 0.74) ^a^	0.00	19%
Special care nursery or neonatal intensive care nursery admission for hypoglycaemia	RCT: 1	76	RR 11.00 (0.63, 192.24) ^a^	0.10	
	Cohort: 1	52	OR 0.06 (0.00, 1.08) ^a^	0.06	-
Stillbirth/neonatal death	Cohort: 1	100	OR 0.28 (0.04, 1.76) ^a^	0.17	-
Apgar score < 7 at 5 min	Cohort: 1	853	OR 0.39 (0.20, 0.76) ^a^	0.01	-
Respiratory distress syndrome	Cohort: 1	100	OR 0.08 (0.00, 1.82) ^a^	0.11	-
Breastmilk feeding exclusively from birth to discharge	RCT: 1	76	RR 0.81 (0.51, 1.28) ^a^	0.36	-
Duration of Initial hospital stay	Cohort: 1	53	MD 0.00 (-3.36, 3.36) days ^a^	1.00	-
Adverse effects*	Cohort: 1	263	OR 0.61 (0.37, 1.03) ^a^	0.06	-
	Case-control: 1	261	MD -0.35 (-0.70, 0.00) mmol/L^b^	0.05	-
Developmental impairment at follow-up	Cohort: 1	131	OR 1.26 (0.58, 2.73) ^a^	0.56	-
**Maternal Outcomes**					
Glycaemic control achieved	RCT: 1	76	RR 0.60 (0.44, 0.81) ^a^	0.00	-
	Cohort: 1	52	OR 2.98 (0.87, 10.16) ^a^	0.08	-
	Case-control: 1	3,680	OR 0.41 (0.32, 0.53)^c^	0.00	-
Use of additional intrapartum pharmacological treatment for maternal glycaemic control	RCT: 1	76	RR 12.00 (1.64, 87.77) ^a^	0.01	-
	Cohort: 3	1,701	OR 0.22 (0.03, 1.68) ^a^	0.15	83%
	Case-control: 1	410	OR 1.46 (0.73, 2.91) ^c^	0.28	23%
Duration of labour	RCT: 1	76	MD -0.02 (-1.39, 1.35) hours ^a^	0.98	-
	Cohort: 1	129	MD -3.30 (-5.86, -0.74) hours ^a^	0.01	-
Mode of birth (caesarean section)	RCT: 1	76	RR 0.78 (0.32, 1.87) ^a^	0.58	-
	Cohort: 4	1,759	OR 1.62 (1.10, 2.39) ^a^	0.01	35%
Postpartum haemorhage	Cohort: 1	100	OR 0.15 (0.01, 3.69) ^a^	0.24	-

**RCT**: randomised controlled trial; **CI**: confidence interval; **MD**: mean difference; **OR**: odds ratio; **RR**: risk ratio; **P-value**: Probability-value

^**a**^ Comparison between tighter intrapartum glycaemic control and less tight glycaemic control

^**b**^ Comparison of maternal blood glucose concentrations for selected outcome

^**c**^ Comparison between neonatal hypoglycaemia and no neonatal hypoglycaemia for selected outcome

* Adverse effects were defined in the cohort study as respiratory distress syndrome, hyperbilirubinemia, perinatal death, shoulder dystocia, and hypoglycaemia requiring treatment, and in the case-control study as respiratory disorders, feeding problems, symptomatic hypoglycaemia, bilirubinaemia requiring treatment and polycythaemia (haematocrit > 70%)

#### Maternal

In women managed using tight glycaemic control compared to less tight/no glycaemic control, the RCT reported no effect on glycaemic control achieved, whereas evidence from two observational studies reported an associated increase in achieved glycaemic control, but the evidence is very uncertain. The RCT also reported no effect on additional intrapartum pharmacological treatment and caesarean section, whereas the observational studies reported an increase for both these outcomes, although the evidence is very uncertain [Additional File 4, Figure S4 and S5]. The evidence is very uncertain about the effect of intrapartum glycaemic control on the duration of labour, with the RCT reporting decreased duration of labour and one observational study reporting an increase. One cohort study reported that tight intrapartum glycaemic control may be associated with an increase in post-partum haemorrhage, but the evidence is very uncertain (Table 1).

### B: Women without diabetes

For women without diabetes, three cohort studies reported outcomes of interest, with only one providing data. The evidence from this one study is very uncertain about the effect of tight glycaemic control on neonatal hypoglycaemia (one study, 30 infants, OR 148.20 (6.45, 3402.92), *p* = 0.002) and duration of labour (one study, 30 infants, MD 1.20 (-0.68, 3.08), *p* = 0.21) [50]. Although data were unable to be used in meta-analysis, Hussein 2014 reported that maternal blood glucose concentrations at delivery were negatively associated with infant blood glucose concentrations at 2 h of age. There was no difference in maternal blood glucose concentrations between those who birthed by caesearean section or vaginally [51]. Additionally, one study reported that there was a significant correlation (*p* < 0.05) between maternal blood glucose concentrations at delivery > 6.67mmol/L and the chance of their infant developing low blood glucose concentrations [52].

### Subgroup analyses

#### Type of diabetes

**Fig. 4:**
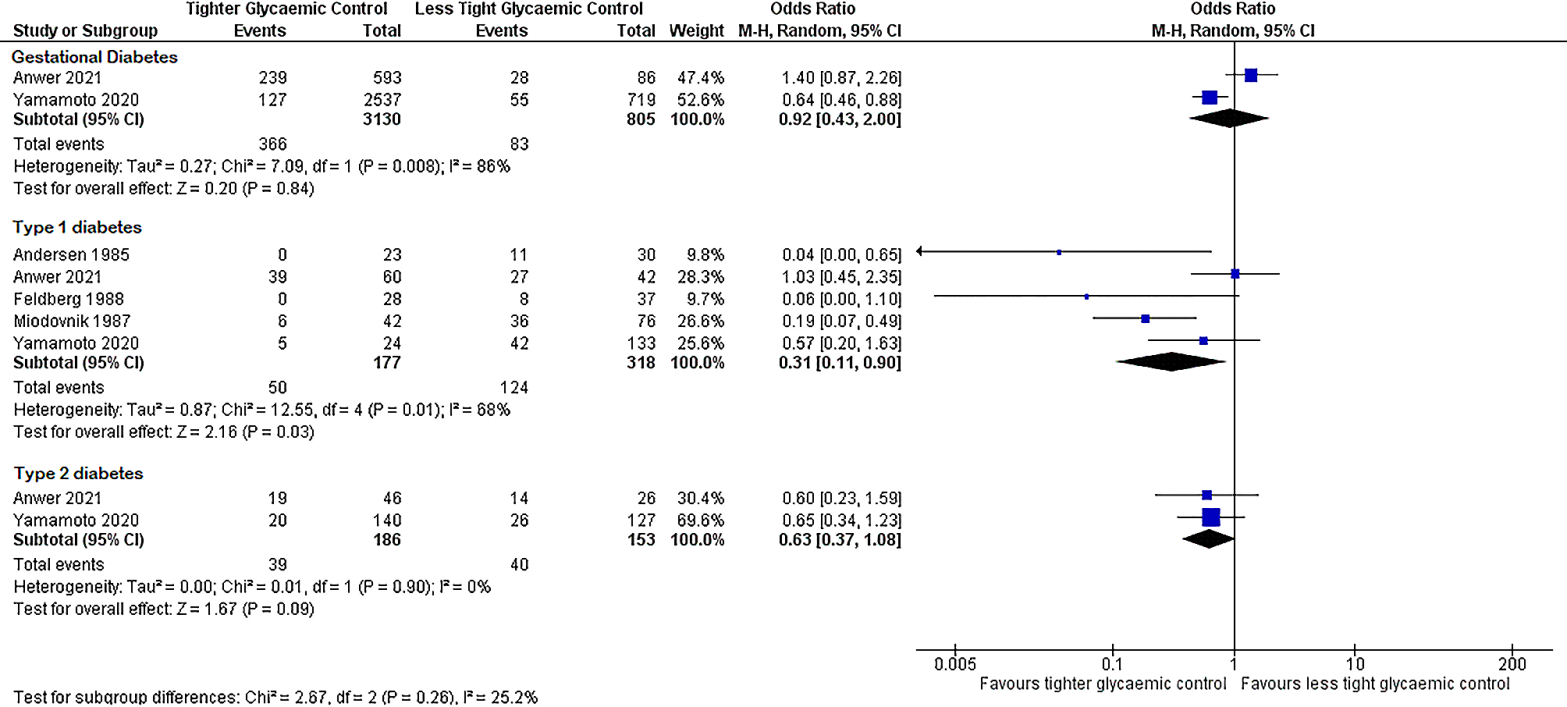
Tighter versus less tight intrapartum glycaemic control in women with gestational, type 1 and type 2 diabetes

We found no significant interaction between type of diabetes and the association between intrapartum maternal glycaemic control and the incidence of neonatal hypoglycaemia (*p* = 0.26 for interaction, Fig. 4).

Insufficient data were available for other planned subgroup analyses.

### Certainty of evidence (GRADE assessment)

#### Neonatal outcomes

For women with diabetes the certainty of the evidence for neonatal hypoglycaemia was assessed as low (RCT) and very low (observational studies). The certainty of evidence was assessed as low for the outcomes of treatment for hypoglycaemia (RCT and observational studies) and breastmilk feeding exclusively (RCT). The certainty of the evidence was assessed as very low for special care nursery or neonatal intensive care nursery admission for hypoglycaemia (RCT and observational study), duration of hospital stay (observational study), and developmental impairment at follow-up (observational study) (Table 2). For women without diabetes, the certainty of evidence was assessed as very low for neonatal hypoglycaemia (observational study) (Table 3).

**Table 2:** Certainty of evidence (GRADE assessment) for neonatal and maternal outcomes for women with diabetes

Outcomes	Number of participants (studies) Follow-up	Certainty of the evidence (GRADE)	Relative effect (95% CI)	Anticipated absolute effects	
				Risk with standard care	Risk difference with tighter control
Neonatal hypoglycaemia	76 (1 RCT)	⨁⨁◯◯ Low^a^	**RR 1.00** (0.45 to 2.24)	237 per 1,000	**0 fewer per 1,000** (130 fewer to 294 more)
	6,152 (11 observational studies)	⨁◯◯◯ Very low^b, c^	**OR 0.44** (0.31 to 0.63)	225 per 1,000	**112 fewer per 1,000** (136 fewer to 68 fewer)
Receipt of treatment for hypoglycaemia during initial hospital stay	76 (1 RCT)	⨁⨁◯◯ Low^a^	**RR 1.13** (0.67 to 1.92)	368 per 1,000	**48 more per 1,000** (122 fewer to 339 more)
	3,785 (3 observational studies)	⨁⨁◯◯ Low^b^	**OR 0.38** (0.18 to 0.80)	136 per 1,000	**80 fewer per 1,000** (83 fewer to 55 fewer)
Special care nursery or neonatal intensive care nursery admission for hypoglycaemia	76 (1 RCT)	⨁◯◯◯ Very low^d^	**RR 11.00** (0.63 to 192.24)	0 per 1,000	**0 fewer per 1,000** (0 fewer to 0 fewer)
	52 (1 observational study)	⨁◯◯◯ Very low^a, b^	**OR 0.06** (0.00 to 1.08)	267 per 1,000	**245 fewer per 1,000** (267 fewer to 15 more)
Breastmilk feeding exclusively from birth to discharge	76 (1 RCT)	⨁⨁◯◯ Low^a^	**RR 0.81** (0.51 to 1.28)	553 per 1,000	**105 fewer per 1,000** (271 fewer to 155 more)
Duration of initial hospital stay	53 (1 observational study)	⨁◯◯◯ Very low^a, e^	-	The mean duration of initial hospital stay was **4.67** days	**0 days** (0 to 0)
Developmental impairment at follow-up	131 (1 observational study)	⨁◯◯◯ Very low^f^	**OR 1.26** (0.58 to 2.73)	359 per 1,000	**55 more per 1,000** (114 fewer to 246 more)
Maternal Hypoglycaemia	76 (1 RCT)	⨁⨁◯◯ Low^a^	**RR 2.00** (0.54 to 7.42)	79 per 1,000	**79 more per 1,000** (36 fewer to 507 more)
	52 (1 observational study)	⨁◯◯◯ Very low^a, b^	**OR 0.13** (0.01 to 2.56)	133 per 1,000	**114 fewer per 1,000** (132 fewer to 149 more)
Glycaemic control achieved	76 (1 RCT)	⨁⨁◯◯ Low^g^	**RR 0.60** (0.44 to 0.81)	921 per 1,000	**368 fewer per 1,000** (516 fewer to 175 fewer)
	52 (1 observational study)	⨁◯◯◯ Very low^a, b^	**OR 2.98** (0.87 to 10.16)	533 per 1,000	**240 more per 1,000** (35 fewer to 387 more)
Use of additional intrapartum pharmacological treatment for maternal glycaemic control	76 (1 RCT)	⨁⨁◯◯ Low^g^	**RR 12.00** (1.64 to 87.77)	26 per 1,000	**289 more per 1,000** (17 more to 2,283 more)
	1,582 (3 observational studies)	⨁◯◯◯ Very low^b, h^	**OR 0.22** (0.03 to 1.68)	236 per 1,000	**172 fewer per 1,000** (229 fewer to 70 fewer)
Mode of birth	76 (1 RCT)	⨁⨁◯◯ Low^a^	**RR 0.78** (0.32 to 1.87)	237 per 1,000	**52 fewer per 1,000** (161 fewer to 206 more)
	1,082 (3 observational studies)	⨁⨁◯◯ Low	**OR 1.62** (1.10 to 2.39)	314 per 1,000	**112 more per 1,000** (21 fewer to 208 more)

**CI**: confidence interval; **MD**: mean difference; **OR**: odds ratio; **RCT**: randomised controlled trial; **RR**: risk ratio

Explanations

a. Downgraded one level for imprecision due CI including both benefits and harm and one level for indirectness for a single study with small sample size

b. Downgraded one level for risk of bias due to quality assessment resulting in a spread of studies being weak or moderate

c. Downgraded one level for inconsistency due to substantial heterogeneity

d. Downgraded two levels for imprecision due wide CI including both benefits and harm and one level for indirectness for a single study with small sample size

e. Downgraded two levels for risk of bias due to quality assessment rating of weak for the study

f. Downgraded one level for imprecision due to CI including both benefits and harm

g. Downgraded one level for imprecision due to low event rate and one level for indirectness for a single study with small sample size

h. Downgraded two levels for inconsistency due to considerable heterogeneity

#### Maternal outcomes

For women with diabetes, the certainty of evidence for glycaemic control achieved was assessed as low (RCT) and very low (observational study). For hypoglycaemia, the certainty of evidence was assessed as low (RCT) and very low (observational study). For the use of additional intrapartum pharmacological treatment, the certainty of evidence was assessed as low (RCT) and very low (observational studies). For mode of birth, the certainty of evidence was assessed as low (RCT and observational studies) (Table 2). For women without diabetes, no data were available for any GRADE outcomes.

**Table 3:** Certainty of evidence (GRADE assessment) for neonatal outcomes for women without diabetes

Outcomes	Number of participants (studies) Follow-up	Certainty of the evidence (GRADE)	Relative effect (95% CI)	Anticipated absolute effects	
				Risk with standard care	Risk difference with tighter control
Neonatal hypoglycaemia	30 (1 observational study)	⨁◯◯◯ Very low^a, b^	**OR 148.20** (6.45 to 3402.92)	0 per 1,000	**0 fewer per 1,000** (0 fewer to 0 fewer)

**CI**: confidence interval; **OR**: odds ratio

Explanations

a. Downgraded one level for risk of bias due to moderate quality assessment rating of the study

b. Downgraded three levels for imprecision due to small sample size and wide CI

## Discussion

### Summary of main results

In principle, tighter intrapartum maternal glycaemic control may reduce neonatal hypoglycaemia because high or variable maternal glucose concentrations may stimulate fetal pancreatic beta cells, leading to fetal hyperinsulinaemia. Once the infant is born, these high insulin concentrations, in the absence of an adequate glucose supply, can lead to hypoglycaemia [53]. Tight maternal intrapartum glycaemic control may decrease the supply of glucose across the placenta to the fetus and thus reduce the likelihood of neonatal hypoglycaemia [53, 54].

The evidence from this systematic review suggests that tight intrapartum maternal glycaemic control compared with less tight or no glycaemic control may be associated with reduced risk of neonatal hypoglycaemia. Although the one included RCT did not find a relationship between tighter intrapartum glycaemic control and neonatal hypoglycaemia, the majority of observational studies did find that tighter intrapartum glycaemic control may be associated with reduced risk of neonatal hypoglycaemia, and also with a decrease in severe hypoglycaemia, admission to special care nursery or neonatal intensive care nursery and adverse effects, with no change in duration of initial hospital stay. However, the evidence is very uncertain because it is derived from only one small RCT and there was heterogeneity within the observational studies with almost half rated weak for methodology overall.

With tighter intrapartum glycaemic control, there is the risk of maternal hypoglycaemia due to limited maternal intake and use of insulin [55]. We found that tighter intrapartum maternal glycaemic control may increase maternal hypoglycaemia, although only three of the 51 studies provided data on this outcome.

In addition, the evidence suggests tighter intrapartum glycaemic control is associated with an increase in use of additional pharmacological treatment and caesarean section delivery. Intrapartum glycaemic control usually involves intravenous or subcutaneous infusion of insulin [19, 56] which requires trained staff, frequent monitoring, glucose and insulin therapy and equipment such as glucometers and infusion pumps which results in cost to the health sector [19, 57]. Financial analysis of the cost of intrapartum glycaemic control is yet to be undertaken. However, one study included in this review stated that implementation of a redesigned care delivery package which incorporated both management during pregnancy and an intrapartum glycaemic control intervention reduced mean payer neonatal reimbursements by over $18,000USD per birth, and for every 10 days of the new programme, the mean neonatal intensive care unit length of stay decreased by 1 day [58]. Thus, the costs of additional maternal care may be balanced by the reduced costs of care for their infants, particularly those who develop neonatal hypoglycaemia.

Recommendations about intrapartum glycaemic control may differ for women with different types of diabetes. In subgroup analysis we did not find that the association between glycaemic control and neonatal hypoglycaemia differed in women with different types of diabetes. However, this analysis included only two cohort studies each for women with gestational and type 2 diabetes, so our sample size and ability to draw a conclusion regarding the difference in diabetes type for this outcome was limited.

We undertook a separate analysis for women without diabetes. During active labour, glucose production rises due to raised hormones such as catecholamines, cortisol and glucagon [59]. Additionally, there is an increased demand for glucose with insulin concentrations suppressed during labour, balancing the supply of and demand for glucose [20]. However, interventions such as administration of antenatal corticosteroids or an intravenous glucose infusion during caesarean section may alter maternal blood glucose concentrations. In light of this, we searched for evidence about the association between intrapartum glycaemia and neonatal hypoglycaemia in women without diabetes. We found insufficient evidence for any of the outcomes of interest in women without diabetes, perhaps in part because intrapartum glucose concentrations are rarely measured in these women.

Our findings are similar to those of an earlier systematic review, which compared the association between in-target (glucose 4.0-7.0mmol/L) versus out-of-target intrapartum maternal glycaemic control and the incidence of neonatal hypoglycaemia. That review reported that this association varied, with 12 of 23 studies reporting no association and six showing a positive relationship between in-target intrapartum control and neonatal hypoglycaemia [22]. The studies included in the previous systematic review also provided low-quality evidence, and the authors concluded that higher quality studies are needed before definitive conclusions can be drawn. Despite our inclusion of an additional 28 studies, this conclusion remains appropriate.

One possible reason for the heterogeneity observed for some outcomes may be the variation in both target and achieved intrapartum glucose concentrations. In the RCT, only the upper limit of the target range differed between groups (3.9-5.6mmol/L in the tight group and 3.9-6.7mmolL in the less tight group) and the median achieved glucose concentration was similar in both groups (5.3mmol/L in the tight group and 5.4mmol/L in the less tight group). Thus, the lack of effect on neonatal hypoglycaemia reported in this RCT may have been due to the minimal differences between the groups in achieved maternal glycaemic control. The 11 cohort studies also used a variety of intrapartum glycaemic target ranges, and often compared groups with maternal glucose concentrations within versus above a target range. There was also variability in the definition of neonatal hypoglycaemia, with many studies using a specific glucose concentration (all < 2.6mmol/L) but some using requirement for intravenous dextrose treatment as the definition.

Single studies appeared to contribute to heterogeneity in some analyses. In the cohort meta-analysis, the observed heterogeneity for the outcome of neonatal hypoglycaemia was primarily due to one specific study (Anwer 2021 [36]). In this study, some participants had glucose measurements taken more than 2 h before birth, and these were more commonly in the tight glycaemic control group, whereas the protocol specified glucose measurements every 1–2 h during labour. This may have contributed to the findings of no difference in neonatal hypoglycaemia between groups, in contrast to all other cohort studies. In the case control meta-analysis, one study (Flores-Le Roux 2010 [37]) appeared to be the main source of heterogeneity, as it was the only study that showed a greater risk of neonatal hypoglycemia with lower maternal glucose concentrations, in contrast to all other case control studies. We did not find any explanation for this difference.

### Strengths and limitations of this review

A strength of this systematic review was the strong methodological approach which involved a pre-registered protocol, broad search strategy, and use of standardised tools for evidence evaluations and statistical analysis. The included sample size was large (11,273 infants in 51 studies), and studies were conducted in 18 countries, suggesting that the conclusions may be applicable across a variety of cultural settings, at least in high-income countries.

However, limited data were available for most of the outcomes of interest, and most planned sensitivity analyses were not possible for this reason. In addition, seven studies were reported only in abstract format and with very limited detail available. There was also evidence of publication bias for some analyses. Only one study was carried out in a low-income country, potentially limiting the applicability of the findings to these regions.

### Implications for future research and practice

We found weak evidence that intrapartum tighter glycaemic control may decrease the incidence of neonatal hypoglycaemia, with other associated neonatal benefits, although this may increase the incidence of intrapartum maternal hypoglycaemia. However, caution is required in interpretation of these outcomes due to the low to very low certainty in GRADE assessment. The optimal targets for tight glycaemic control and the balance of risks and benefits for the infant and mother are still unclear. More high-quality RCTs are required that report on achieved as well as target maternal glycaemia, report adverse effects for both mother and infant, and provide information about women with different types of diabetes and women without diabetes. Measures of cost effectiveness and acceptability of the intervention are also required.

### Electronic supplementary material

Below is the link to the electronic supplementary material.

Supplementary Material 1

Supplementary Material 2

Supplementary Material 3

Supplementary Material 4

## Data Availability

The datasets used and/or analysed during the current study are available from the corresponding author on reasonable request.

## Abbreviations

RCT: Randomised controlled trial
CENTRAL: Cochrane Central Register of Controlled Trials
WHO: World Health Organisation
ICTRP: International Clinical Trials Registry Platform
EPHPP: Effective Public Health Practice Project
RoB: Cochrane Risk of Bias tool
GRADE: Grading of Recommendations Assessment, Development and Evaluation
GDT: Grade Pro Guideline Development Tool
RR: Relative risk
OR: Odds ratio
MD: Mean difference
CI: Confidence interval
GDM: Gestational diabetes mellitus
T1DM: Type 1 diabetes mellitus
T2DM: Type 2 diabetes mellitus
