# Supplementary material for: Intrapartum maternal glycaemic control for the prevention of neonatal hypoglycaemia: a systematic review and meta-analysis
Source: BMC Pregnancy Childbirth. 2024 Jun 13;24:423. doi: 10.1186/s12884-024-06615-8 (PMC11170869; doi:10.1186/s12884-024-06615-8)
Supplement: Supplementary file 1 — Supplementary Material 1 [file 12884_2024_6615_MOESM1_ESM.docx]

## Search Strategy

**Appendix S1. Literature search, last performed on the 30^th^ of November 2023 using Medline, Embase, CINAHL COMPLETE and Cochrane CENTRAL. Search was also completed on Australian and New Zealand Clinical Trials Registry (**[**https://www.anzctr.org.au/**](https://www.anzctr.org.au/)**), Clinical Trials (**[**www.ClinicalTrials.gov**](http://www.clinicaltrials.gov/)**) and the World Health Organisation (WHO) International Clinical Trials Registry Platform (ICTRP) Search Portal (**[**https://apps.who.int/trialsearch/**](https://apps.who.int/trialsearch/)**)**

**Ovid MEDLINE**

1 exp infant, newborn/ or Intensive Care, Neonatal/ or Intensive Care Units, Neonatal/

2 (babe or babes or baby* or babies or infant? or infantile or infancy or neonat* or neo-nat* or newborn* or new born? or newly born or premature or pre-mature or pre-matures or prematures or prematurity or pre-maturity or preterm or preterms or pre term? or preemie or preemies or premies or premie or NICU or NICUs).ti,ab,kw,kf.

3 1 or 2

4 delivery, obstetric/ or cesarean section/ or exp extraction, obstetrical/ or labor, induced/ or vaginal birth after cesarean/

5 Glycemic Control/

6 Blood Glucose/

7 5 or 6

8 (birth* or childbirth or intrapartum or labo?r or parturition or intra partum or c?esarean or (obstetric* adj2 extract*)).mp.

9 4 or 8

10 7 and 9

11 ((glyc?emi* or glucose or sugar) adj5 (birth* or childbirth or intrapartum or labo?r or deliver* or parturition or intra partum or c?esarean or (obstetric* adj2 extract*))).mp.

12 10 or 11

13 3 and 12

14 limit 13 to "humans only (removes records about animals)"

**EMBASE (1980- )**

1 exp infant, newborn/ or Intensive Care, Neonatal/ or Intensive Care Units, Neonatal/

2 (babe or babes or baby* or babies or infant? or infantile or infancy or neonat* or neo-nat* or newborn* or new born? or newly born or premature or pre-mature or pre-matures or prematures or prematurity or pre-maturity or preterm or preterms or pre term? or preemie or preemies or premies or premie or NICU or NICUs).ti,ab,kw,kf.

3 1 or 2

4 Glycemic Control/

5 Blood Glucose/

6 4 or 5

7 delivery, obstetric/ or cesarean section/ or exp extraction, obstetrical/ or labor, induced/ or vaginal birth after cesarean/

8 (birth* or childbirth or intrapartum or labo?r or parturition or intra partum or c?esarean or (obstetric* adj2 extract*)).mp.

9 7 or 8

10 6 and 9

11 ((glyc?emi* or glucose or sugar) adj5 (birth* or childbirth or intrapartum or labo?r or deliver* or parturition or intra partum or c?esarean or (obstetric* adj2 extract*))).mp.

12 10 or 11

13 3 and 12

14 limit 13 to "humans only (removes records about animals)"

**CINAHL COMPLETE**

S1 Blood Glucose/ OR Glyc#mic Control/Expanders - Apply equivalent subjects

S2 ( (babe or babes or baby* or babies or infant# or infantile or infancy or neonat* or neo-nat* or newborn* or new born# or newly born or premature or pre-mature or pre-matures or prematures or prematurity or pre-maturity or preterm or preterms or pre term# or preemie or preemies or premies or premie or NICU or NICUs) ) OR ( exp infant, newborn/ or Intensive Care, Neonatal/ or Intensive Care Units, Neonatal/ )Expanders - Apply equivalent subjects

S3 ( delivery, obstetric/ or c#sarean section/ or exp extraction, obstetrical/ or lab#r, induced/ or vaginal birth after c#sarean/ ) OR ( (birth* or childbirth or intrapartum or labo#r or parturition or intra partum or c#esarean or (obstetric*N2 extract*)) )Expanders - Apply equivalent subjects

S4 ((glyc#mi* or glucose or sugar) N5 (birth* or childbirth or intrapartum or labo#r or deliver* or parturition or intra partum or c#esarean or (obstetric* N2 extract*)))Expanders - Apply equivalent subjects

S5 (((glyc#mi* or glucose or sugar) N5 (birth* or childbirth or intrapartum or labo#r or deliver* or parturition or intra partum or c#esarean or (obstetric* N2 extract*)))) OR (S1 AND S3)Expanders - Apply equivalent subjects

S6 S2 AND S5 Expanders - Apply equivalent subjects

**Cochrane CENTRAL**

#1 MeSH descriptor: [Glycemic Control] explode all trees

#2 "blood glucose control"

#3 "glucose control"

#4 #1 OR #2 OR #3

#5 MeSH descriptor: [Blood Glucose] explode all trees

#6 "blood sugar"

#7 glyc?mia

#8 #5 OR #6 OR #7

#9 #8 OR #4

#10 MeSH descriptor: [Infant, Newborn] explode all trees

#11 babe or babes or baby* or babies or infant? or infantile or infancy or neonat* or neo-nat* or newborn* or new born? or newly born or premature or pre-mature or pre-matures or prematures or prematurity or pre-maturity or preterm or preterms or pre term? or preemie or preemies or premies or premie or NICU or NICUs

#12 "intensive care" OR "intensive care unit*"

#13 #10 or #11 OR #12

#14 MeSH descriptor: [Labor, Obstetric] explode all trees

#15 MeSH descriptor: [Delivery, Obstetric] explode all trees

#16 (birth* or childbirth or intrapartum or labo?r or parturition or intra partum or c?esarean or (obstetric*NEAR/2 extract*))

#17 #16 OR #15 OR #14

#18 #17 and #9

#19 ((glyc?emi* or glucose or sugar) NEAR/5 (birth* or childbirth or intrapartum or labo?r or deliver* or parturition or intra partum or c?esarean or (obstetric* NEAR/2 extract*)))

#20 #18 OR #19

#21 #20 AND #13
