# Supplementary material for: Intrapartum maternal glycaemic control for the prevention of neonatal hypoglycaemia: a systematic review and meta-analysis
Source: BMC Pregnancy Childbirth. 2024 Jun 13;24:423. doi: 10.1186/s12884-024-06615-8 (PMC11170869; doi:10.1186/s12884-024-06615-8)
Supplement: Supplementary file 3 — Supplementary Material 3 [file 12884_2024_6615_MOESM3_ESM.docx]

## Table S2: Table of characteristics

| **Author and Year** | **Country, Study date** | **Participants** | **Tighter intrapartum glycaemic control** | **Less tight/no intrapartum glycaemic control** | **Outcomes** |
| --- | --- | --- | --- | --- | --- |
| **Randomised controlled trials** | | | | | |
| Hamel 2019 (1) | America,  February 2016 to April 2018 | Inclusion criteria: maternal age ≥18 years, diagnosis of GDM, singleton, no major fetal anomalies, fluency in English or Spanish.  Exclusion criteria: pre-GDM, multiple gestations, major fetal anomaly anticipated to require NICU admission, planned caesarean delivery, HbA1c ≥6.5% | N= 38  Target: 3.9-5.6 mmol/L  Monitoring: Hourly  Timing: Intrapartum | N=38  Target: 3.9-6.7 mmol/L  Monitoring: 4 hourly  Timing: Intrapartum | - Blood glucose < 40 mg/dL within the first 24 h after birth - Maternal hypoglycaemia - Received any intervention for hypoglycaemia (oral, IV, or both)* - Admission to NICU - NICU admission for hypoglycaemia - Breastmilk feeding - 5-min Apgar score ≥ 9 - Insulin for maternal hyperglycaemia - Spontaneous vaginal delivery - Labour duration |
| **Prospective Cohort studies** | | | | | |
| Cahill 2016 (1) | America, June 2008 to April 2012 | Inclusion criteria: diagnosis of T2DM or GDM  Exclusion criteria: multiple gestation, fetus with known congenital anomalies, T1DM, no English | N= 90  Target: ≤6.8 mmol/L  Monitoring: NS  Timing: admission to labour and delivery | N= 173  Target: >6.8 mmol/L  Monitoring: NS  Timing: at delivery | - Composite neonatal morbidity |
| Djomhou 2016 (2) | Cameroon,  March to June 2013 | Inclusion criteria: diabetic, women who had fasted for 8 to 12 h, consented to participate.  Exclusion criteria: overt diabetes | N= 69  Target: < 5.1 mmol/L  Monitoring: NS  Timing: during labour | N= 69  Target: ≥5.1 mmol/L  Monitoring: NS  Timing: during labour | - Caesarean section - Vaginal delivery - Post-partum haemorrhage - Intrauterine death - Respiratory distress - Rapid neonatal death - Intrapartum death |
| Flores-LeRoux 2010 (3) | Spain,October 2006 to March 2009 | Inclusion criteria: GDM  Exclusion criteria: Multiple pregnancy | N= 111  Target: <7.2 mmol/L  Monitoring: 2 hourly  Timing: last measurement before delivery | N= 18  Target: >7.2 mmol/L  Monitoring: 2 hourly  Timing: last measurement before delivery | - Labour duration - Elective caesarean section |
| Griffith 2019 (4) | New Zealand, December 2006 to November 2010 | Inclusion criteria: infants at risk of neonatal hypoglycaemia (maternal diabetes, late preterm birth between 32 and 36 weeks' gestation, SGA, LGA) | N= 92  Target: In range (4.0-7.0 mmol/L)  Monitoring: NS  Timing: in 6 hours prior to birth | N= 39  Target: Out of range (<4.0 or >7.0 mmol/L)  Monitoring: NS  Timing: in 6 hours prior to birth | - Neurosensory impairment at 4.5 years of age |
| Miodovnik 1987 (5) | America,  1978 | Inclusion criteria: diabetic mothers | N= 42  Target: maximum <5.0 mmol/L  Monitoring: hourly  Timing: during labour | N= 76  Target: maximum >5.0 mmol/L  Monitoring: hourly  Timing: during labour | - Plasma glucose concentration < 1.7mmol/L in first 4 h after birth |
| Omene 1979 (6) | Nigeria, NS | Inclusion criteria: spontaneous delivery, Apgar =>7  Exclusion criteria: low birth weight, infants of toxaemic or diabetic mothers | N= 11  Target: <3.3 mmol/L  Monitoring: NS  Timing: at end of second stage of labour | N= 19  Target: >3.9 mmol/L  Monitoring: NS  Timing: at end of second stage of labour | - Blood glucose < 1.7mmol/L in first 72h after birth - Duration of labour |
| Soler 1978 (7) | England, NS | Inclusion criteria: insulin dependent diabetic | N= 31  Target: 5-7.2 mmol/L  Monitoring: NS  Timing: during labour and at delivery | N= 44  Target: >7.2 mmol/L  Monitoring: NS  Timing: during labour and at delivery | - Neonatal hypoglycaemia |
| Syrop 2021 (8) | America, November 2015 to June 2018 | Inclusion criteria: insulin-requiring diabetes in pregnancy, between 6 and 29 6/7 weeks gestation at perinatal diabetes care registration, received ongoing care, cellular-enabled glucometers  Exclusion criteria: multiple gestation | N= 93  Target: <7.8 mmol/L  Monitoring: NS  Timing: during delivery | N= 26  Target: ≥7.8 mmol/L  Monitoring: NS  Timing: during delivery | - NICU admission |
| **Retrospective cohort studies** | | | | | |
| Andersen 1985  (1) | Denmark,  1977-1983 | Inclusion criteria: insulin dependent diabetic mothers, recorded plasma glucose concentration at birth and at two hours old, no calories supplied to infant | N= 53  Target: 4.4-6.4 mmol/L  Monitoring: NS  Timing: during delivery | N= 53  Target: 7.1-14.8 mmol/L  Monitoring: NS  Timing: during delivery | - Neonatal hypoglycaemia - IV glucose treatment to infants with glucose values ≤ 1.7 mmol/L |
| Anwer 2021 (2) | America, January 2017 to June 2019 | Inclusion criteria: Singleton, GDM or pre-GDM  Exclusion criteria: multiple gestation, intrauterine fetal demise, pre-viable birth | N= 699  Target: ≤6.1 mmol/L  Monitoring: every 2–4 h (early labour) and 1–2 h (active labour)  Timing: labour | N=154  Target: >6.1 mmol/L  Monitoring: every 2–4 h (early labour) and 1–2 h (active labour)  Timing: labour | - Neonatal hypoglycaemia at 2-24h - NICU admission - Apgar ≤7 at 5 min - Mode of delivery - IV insulin   Adjustment for maternal age, race/ethnicity, body mass index, IV insulin, insurance status, and diabetes type. |
| Dashora 2017 (3) | United Kingdom, July 2014 to June 2015 | Inclusion criteria: diabetes mellitus | N=26  Target: 4.0–7.0 mmol/L  Monitoring: NS  Timing: once in labour | N=9  Target: >7.0 mmol/L  Monitoring: NS  Timing: once in labour | - Neonatal hypoglycaemia |
| Fleischman 2020 (4) | America, April 2014 to March 2017 | Inclusion criteria: GDM or pre-GDM, liveborn, singleton at ≥ 24 weeks’ gestation at a tertiary academic centre | N=241  Target: Tight < 6.1 mmol/L, moderately tight 6.1-7.7 mmol/L  Monitoring: NS  Timing: last measurement prior to delivery | N=22  Target: ≥7.8 mmol/L  Monitoring: NS  Timing: last measurement prior to delivery | - Neonatal hypoglycaemia   Adjustment for maternal age, BMI, race, nulliparity, type of diabetes, chronic hypertension, gestational hypertension or preeclampsia, GA at delivery, sex  and LGA |
| Heo 2018 (5) | America, NS | Inclusion criteria: GDM  Exclusion criteria: Pre-GDM; caesarean delivery scheduled; precipitous deliveries | N=22  Target: 3.9-5.6 mmol/L  Monitoring: NS  Timing: when admitted for labour | N= 30  Target: 3.9-5.6 mmol/L  Monitoring: NS  Timing: when admitted for labour | - NICU admission - NICU admission for hypoglycaemia - Maternal hypoglycaemia - Glucose within target in last 6h before birth - Insulin infusion during labour |
| Kline 2007 (6) | Canada,NS | Inclusion criteria: T1DM, T2DM, live birth.  Exclusion criteria: GDM, missing/incomplete data, delivery at other institutions | N= 45  Target: < 7.0 mmol/L  Monitoring: 2 hourly (active labour)  Timing: at delivery | N= 55  Target: > 7.0 mmol/L  Monitoring: 2 hourly (active labour)  Timing: at delivery | - Neonatal hypoglycaemia |
| McCarley 2023 (7) | America, September 2018 to July 2022 | Inclusion criteria: CGM during pregnancy, pre-GDM, livebirth, singleton  Exclusion criteria: major fetal anomalies | N= 42  Target: time in range (3.6 -7.2 mmol/L) >70%  Monitoring: NS  Timing: 6h before delivery | N= 11  Target: time in range (3.6 -7.2 mmol/L) <70%  Monitoring: NS  Timing: 6h before delivery | - Neonatal hypoglycaemia - Receipt of treatment for hypoglycaemia - NICU admission - Length of stay   Adjustment for type of diabetes |
| Nipakakul 2023 (8) | Thailand, October 2016 to September 2019 | Inclusion criteria: Term, singleton, diabetes | N=530  Target: <6.1 mmol/L  Monitoring: NS  Timing: intrapartum | N=147  Target: ≥6.1 mmol/L  Monitoring: NS  Timing: intrapartum | - Neonatal hypoglycaemia - Intrapartum insulin use - Mode of delivery   Adjustment for mode of delivery |
| Roman 2017 (9) | America ,October 2011 to July 2016 | Inclusion criteria: Diabetes, GDM requiring medication  Exclusion criteria: caesarean section scheduled | N=80  Target: 3.3- 5.6 mmol/L  Monitoring: NS  Timing: during the last 6 and 12 h before delivery | N=72  Target: >5.6 mmol/L  Monitoring: NS  Timing: during the last 6 and 12 h before delivery | - Neonatal hypoglycaemia |
| Yamamoto 2020 (10) | Canada, January 2007 to December 2014 | Inclusion criteria: pre-GDM or GDM, multiple gestation pregnancy  Exclusion criteria: delivered outside of the study area or after study period, unclear diabetes type, unknown expected date | N=2701  Target: 3.5-6.5 mmol/L  Monitoring: hourly (active labour)  Timing: intrapartum | N=979  Target: ≥8.6 mmol/L  Monitoring: hourly (active labour)  Timing: intrapartum | - Neonatal hypoglycaemia - Intravenous dextrose therapy   Adjustment for LGA, preterm delivery and infant sex |
| **Case-Control studies** | | | | | |
| Agrawal 2000 (1)  [Observational study] | Australia, January 1998 to 1999 | Inclusion criteria: ≥37  weeks’ gestation, insulin-dependent diabetic, previous GDM, detected GDM | N= 18  Target: 4.0-8.0 mmol/L  Monitoring: 2 hourly  Timing: Intrapartum | N= 20  Target: 4.0-8.0 mmol/L  Monitoring: 2 hourly  Timing: Intrapartum | - Neonatal hypoglycaemia |
| Alrais 2022 (2)  [Retrospective cohort study] | America, March 2012 to May 2018 | Inclusion criteria: insulin treated T2DM, electronic medical records, >23 weeks gestation,  Exclusion criteria: missing data, multiple gestation, intrauterine fetal demise, major anomalies, chronic renal failure, proliferative retinopathy | N= 95  Target: 3.9-5.6 mmol/L  Monitoring: hourly  Timing: during labour | N= 12  Target: 3.9-5.6 mmol/L  Monitoring: hourly  Timing: during labour | - Insulin infusion |
| Balsells 2000 (3)  [Observational study] | Spain, NS | Inclusion criteria: GDM | N= 5  Target: 2.8-6.9 mmol/L  Monitoring: hourly  Timing: intrapartum | N= 80  Target: 2.8-6.9 mmol/L  Monitoring: hourly  Timing: intrapartum | - Neonatal hypoglycaemia |
| Cordua 2013  (4)  [Randomised control study] | Denmark, February 2009 to 2011 | Inclusion criteria: pre-GDM, singleton,  Exclusion criteria: use of real-time CGM, severe mental or psychiatric disorder, diabetic nephropathy, or severe concurrent comorbidity | N= 10  Target: 4.0-7.0 mmol/L  Monitoring: hourly  Timing: intrapartum | N= 17  Target: 4.0-7.0 mmol/L  Monitoring: hourly  Timing: intrapartum | - Neonatal hypoglycaemia |
| Curet 1997 (5)  [Prospective cohort study] | America, NS | Inclusion criteria: singleton , diabetic mothers requiring insulin | N= 38  Target: 3.3-5.0 mmol/L  Monitoring: NS  Timing: From morning of delivery day until delivery. | N= 195  Target: 3.3-5.0 mmol/L  Monitoring: NS  Timing: From morning of delivery day until delivery. | - Neonatal hypoglycaemia |
| Flores-LeRoux 2010 (6) | Spain, October 2006 to March 2009 | Inclusion criteria: GDM  Exclusion criteria: Multiple pregnancies | N= 112  Target: <7.2 mmol/L  Monitoring: 2 hourly  Timing: last measurement taken before delivery | N= 15  Target: >7.2 mmol/L  Monitoring: 2 hourly  Timing: last measurement taken before delivery | - Insulin therapy in labour - Neonatal hypoglycaemia |
| Flores-LeRoux 2012 (7)  [Prospective cohort study] | Spain, January 2009 to June 2011 | Inclusion criteria: GDM, pre-natal care at study institution  Exclusion criteria: Multiple gestation | N= 48  Target: 3.8-7.2 mmol/L  Monitoring: every 1-2h  Timing: peripartum | N= 142  Target: 3.8-7.2 mmol/L Monitoring: every 1-2h  Timing: peripartum | - Neonatal hypoglycaemia - Moderate-severe hypoglycaemia - Maternal insulin use - Maternal hypoglycaemia - Apgar score at 5 minutes |
| Hong 2022 (8)  [Prospective cross-sectional study] | Malaysia, December 2017 to August 2018 | Inclusion criteria: spontaneous labour, scheduled induction or planned caesarean, aged≥18  years, singleton, ≥37  weeks’ gestation  Exclusion criteria: moderate or severe anaemia in pregnancy, major hemoglobinopathy, gross fetal anomaly, inability to consent | N= 165  Target: NS, mean glucose: 6.4 mmol/L  Monitoring: NS  Timing: delivery | N= 835  Target: NS, mean glucose: 6.3 mmol/L  Monitoring: NS  Timing: delivery | - Postpartum haemorrhage |
| Hussein 2014  (9)  [Case-control study] | Sudan, April to June 2012 | Inclusion criteria: ≥37 weeks’ gestation, ≥2500g, Apgar ≥ 8 at 1 and 5 minutes  Exclusion criteria: any medical disorder, pregnancy and childbirth complications, signs of perinatal stress, instrumental delivery, required intensive resuscitation | N= 55  Target: NS, mean glucose: 6.0 mmol/L  Monitoring: NS  Timing: delivery | N= 55  Target: NS, mean glucose: 6.0 mmol/L  Monitoring: NS  Timing: delivery | - Vaginal and caesarean delivery - Newborn blood glucose at 2 hours of age - Maternal glucose at delivery |
| Lean 1990 (10)  [Observational study] | Scotland, 1987 | Inclusion criteria: insulin-treated diabetes, 22-35years old | N= 11  Target: NS, mean glucose: 7.6 mmol/L  Monitoring: NS  Timing: delivery | N= 14  Target: NS, mean glucose: 5.9 mmol/L  Monitoring: NS  Timing: delivery | - Neonatal hypoglycaemia - Treated with IV glucose |
| Lepercq 2008 (11)  [Prospective cohort study] | Paris, 1997 - 2005 | Inclusion criteria: T1DM  Exclusion criteria: Diabetic ketoacidosis | N= 30  Target: 3.4-7.8 mmol/L  Monitoring: hourly  Timing: labour | N= 199  Target: 3.4-7.8 mmol/L  Monitoring: hourly  Timing: labour | - Neonatal hypoglycaemia |
| Norlander 1989 (12)  [Randomised control study] | Sweden,1981 – 1984 | Inclusion criteria: GDM | N= 61  Target: NS, mean glucose: 8.9 mmol/L  Monitoring: NS  Timing: delivery | N= 200  Target: NS, mean glucose: 9.2 mmol/L  Monitoring: NS  Timing: delivery | - Neonatal morbidity |
| Sargent 2015 (13)  [Retrospective cohort study] | America /  2007-2014 | Inclusion criteria: singleton, T1DM managed with CSII or MDI, ≥36 weeks’ gestation  Exclusion criteria: stillbirth, delivery at an outside institution, patients who crossed over modes of insulin management, incomplete data | N= 59  Target: 3.9-6.7 mmol/L  Monitoring: hourly  Timing: within last 90 minutes of delivery | N= 29  Target: 3.9-6.7 mmol/L  Monitoring: hourly  Timing: within last 90 minutes of delivery | - Neonatal hypoglycaemia |
| Sheehan 1986 (14)  [Case-control study] | America, December 1981 to February 1984 | Inclusion criteria: insulin-dependent diabetes | N= 9  Target: NS, mean glucose: 10.2 mmol/L  Monitoring: NS  Timing: delivery | N= 11  Target: NS, mean glucose: 9.4 mmol/L  Monitoring: NS  Timing: delivery | - Neonatal hypoglycaemia - Severity of hypoglycaemia |
| Stenninger 2008 (15)  [Prospective cohort study] | Sweden,  2003 - 2005 | Inclusion criteria: insulin treated pre-GDM or GDM, ≥37 weeks’ gestation, planned vaginal delivery  Exclusion criteria: Glucose monitoring ended due to emergency caesarean section or failed for technical reasons. | N= 5  Target: NS, mean glucose: 7.5 mmol/L  Monitoring: hourly  Timing: 2 h before delivery | N= 10  Target: NS, mean glucose: 5.3 mmol/L  Monitoring: hourly  Timing: 2 h before delivery | - IV glucose treatment |
| Stewart 2019 (16)  [Prospective cohort study] | United Kingdom, NS | Inclusion criteria: T1DM, singleton, experience with masked or real-time CGM, intensive insulin therapy using either MDI or insulin pump therapy.  Exclusion criteria: congenital anomaly, severe respiratory distress. | N=5  Target: 3.9-7.8 mmol/L  Monitoring: CGM  Timing: during 24 h before delivery | N=11  Target: 3.9-7.8 mmol/L  Monitoring: CGM  Timing: during 24 h before delivery | - Blood glucose ≤1mmol/L - Infant treated with IV dextrose |
| Yamamoto 2018 (17)  [Retrospective cohort study] | United Kingdom, NS | Inclusion criteria: 18 to 45 years old, T1DM for >12 month, HbA1c 6.5%–10% (4.8–8.6 mmol/mol), English speaking, e-mail access | N= 18  Target: 3.5-6.5mmol/L  Monitoring: CGM  Timing: during labour | N= 9  Target: 3.5-6.5mmol/L  Monitoring: CGM  Timing: during labour | - Neonatal hypoglycaemia |
| Yamamoto 2019 (18)  [Randomised control study] | Canada, England, Scotland, Spain, Italy, Ireland, and America, March 2013 to 2016 | Inclusion criteria: T1DM, livebirth, singleton, 13 weeks and 6 days’ gestation or less, and HbA1c between 6·5–10·0%, planning for pregnancy to have HbA1c between 7·0–10·0%  Exclusion criteria: Regular CGM user, severe nephropathy or medical conditions, psychiatric illness requiring hospitalisation | N= 9  Target: 3.5-6.5 mmol/L  Monitoring: CGM  Timing: intrapartum | N= 24  Target: 3.5-6.5 mmol/L  Monitoring: CGM  Timing: intrapartum | - Neonatal hypoglycaemia - Treatment with IV dextrose |
| Yamamoto 2020 (19)  [Retrospective cohort study] | Canada, January 2007 to December 2014 | Inclusion criteria: pre-GDM or GDM, multiple gestation pregnancy.  Exclusion criteria: delivered outside of the study area or after study period, unclear diabetes type, unknown expected date | N=275  Target: 3.5-6.5 mmol/L  Monitoring: hourly (active labour)  Timing: intrapartum | N=3405  Target: 3.5-6.5 mmol/L  Monitoring: hourly (active labour)  Timing: intrapartum | - In target glucose |
| Yoeli-Ullman 2020 (20)  [Retrospective cohort study] | Israel,  2012 - 2015 | Inclusion criteria: T1DM, maternal age 18-45 years old, available CGM data, followed in multi-disciplinary high-risk pregnancy clinic  Exclusion criteria: fetal anomalies, delivery at a different hospital. | N= 13  Mean glucose: 6.9 mmol/L  Monitoring: CGM  Timing: last h prior to delivery | N= 19  Mean glucose: 6.0 mmol/L  Monitoring: CGM  Timing: last h prior to delivery | - Neonatal hypoglycaemia |
| **Papers not included in the meta-analysis** | | | | | |
| Barrett 2009 (1)  [Retrospective cohort study] | Australia, August 2001 to July 2004 | Inclusion criteria: GDM or pre-GDM  Exclusion criteria: multiple gestation pregnancy, delivered before 37.1 weeks’ gestation, no maternal blood glucose measurement until after delivery | N=137  Target: 4.0- 8.0 mmol/L  Monitoring: hourly  Timing: collected most immediately preceding delivery |  | - Neonatal hypoglycaemia |
| Drever 2016 (2)  [Retrospective cohort study] | Canada,  2000–2010 | Inclusion criteria: T1DM  Exclusion criteria: unavailable hospital records, not seen throughout pregnancy | N= 105  Target: 4.0-6.0mmol/L  Monitoring: hourly  Timing: Intrapartum |  | - Neonatal hypoglycaemia - Time with blood glucose between 4 and 6mmol/L - NICU admission |
| Ghio 2009 (3)  [Prospective cohort study] | Italy, NS | Inclusion criteria: GDM and pre-GDM, elective C-section | N= 13  Target: 5.0-6.7 mmol/L  Monitoring: CGM  Timing: Intrapartum |  | - Neonatal hypoglycaemia |
| Haigh 1982 (4)  [Prospective cohort study] | London, Ontario, Canada,  1972-1980 | Inclusion criteria: insulin dependent diabetes | N=51  Target: NS  Monitoring: 3 hourly  Timing: Labour |  | - Neonatal hypoglycaemia |
| Hanson 1986 (5)  [Prospective cohort study] | Sweden /  1979-1982 | Inclusion criteria: pre-GDM, received insulin during pregnancy, Stockholm area  Exclusion criteria: GDM. twin pregnancies, congenital malformations | N=52  Target: NS  Monitoring: every 30 min  Timing: delivery |  | - Neonatal hypoglycaemia - Neonatal morbidity |
| Joshi 2017 (6)  [Retrospective cohort study] | Australia /  2009 - 2014 | Inclusion criteria: pre-GDM | N=226 total  Target: 4.0–7.0 mmol/L  Monitoring: hourly  Timing: 8 h prior to delivery | N= NS  Target: 7.0-10 mmol/L  Monitoring: hourly  Timing: 8 h prior to delivery | - Neonatal hypoglycaemia |
| Mediola 1982 (7)  [Retrospective cohort study] | America / NS | Inclusion criteria: received IV glucose before delivery | N = 56  Target: NS  Monitoring: NS  Duration: delivery |  | - Neonatal hypoglycaemia |
| Schmid 1973 (8)  [Cohort study] | Switzerland/ NS | Inclusion criteria: no pregnancy complications; anterior occipital spontaneous delivery, birth between the 38th and 40th week gestation, Apgar 8-10.  Exclusion criteria: any pregnancy or childbirth complications or diseases, maternal administration of glucose, analgesics, narcotics or spasmolytics, fetal acidosis | N=40  Target: NS  Monitoring: NS  Duration: delivery |  | - Neonatal hypoglycaemia |
| Scott 2020 (9)  [Retrospective cohort study] | United Kingdom / NS | Inclusion criteria: T1DM | N= 12  Target: 4.0-8.0 mmol/L  Monitoring: CGM Timing: within 24h before delivery |  | - Neonatal hypoglycaemia - Respiratory distress syndrome - Admission to special care baby unit |
| Stenninger 1991 (10)  [Prospective cohort study] | Sweden / April 1986 to March 1987 | Inclusion criteria: GDM and pre-GDM, Orebro county. | N=36  Target: NS  Monitoring: NS  Timing: during the day of delivery |  | - Neonatal hypoglycaemia |
| Taylor 2002 (11)  [Retrospective cohort study] | United Kingdom / January 1994 to 1999 | Inclusion criteria: T1DM, singleton  Exclusion criteria: multiple birth pregnancy | N= 94  Target: 4.0–8.0 mmol/L  Monitoring: NS  Timing: 9h prior to delivery | N= 13  Target: >8.0 mmol/L  Monitoring: NS  Timing: 9h prior to delivery | - Neonatal hypoglycaemia |
| Tuohy 2021 (12)  [Retrospective cohort study] | New Zealand / January 2006 to December 2016 | Inclusion criteria: GDM or pre-GDM, birth at < 37.0 weeks’ gestation; been to hospital for > 2 hours for any reason during pregnancy (22.0 −36.6 weeks’ gestation), elective caesarean section (any gestation) or emergency caesarean section < 39 weeks’ gestation  Exclusion criteria: no glycaemic data | N= 634  Target: 3.5-7.0 mmol/L  Monitoring: NS  Timing: within 24 h of birth |  | - Neonatal hypoglycaemia - Severe Neonatal hypoglycaemia |
| Yeast 1978 (13)  [Prospective cohort study] | America / NS | Inclusion criteria: diabetes, maintained on CSII during the peripartum period. | N=17 infants/16 mothers  Target: 3.9-7.2 mmol/L  Monitoring: NS  Timing: intrapartum |  | - Neonatal hypoglycaemia |
| Zelivianskaia 2019 (14)  [Retrospective cohort study] | America  2016 - 2017 | Inclusion criteria: GDM or pre-GDM, delivered at an urban institution | N=191  Target: NS  Monitoring: NS  Timing: during labour |  | - Neonatal hypoglycaemia |

**GDM:** gestational diabetes mellitus; **pre-GDM**: pregestational diabetes mellitus; **NICU**: neonatal intensive care unit; **HbA1c:** glycated hemoglobin; **T2DM**: type 2 diabetes mellitus; **T1DM**: type 1 diabetes mellitus; **NS**: not specified; **h**: hours; **SGA**: small for gestational age; **LGA**: large for gestational age; **CGM**: continuous glucose monitoring; **CSII:** continuous subcutaneous insulin infusion; **MDI**: multiple daily injections of insulin; **IV**: intravenous
