# Supplementary material for: Intrapartum maternal glycaemic control for the prevention of neonatal hypoglycaemia: a systematic review and meta-analysis
Source: BMC Pregnancy Childbirth. 2024 Jun 13;24:423. doi: 10.1186/s12884-024-06615-8 (PMC11170869; doi:10.1186/s12884-024-06615-8)
Supplement: Supplementary file 4 — Supplementary Material 4 [file 12884_2024_6615_MOESM4_ESM.docx]

## Secondary outcomes figures


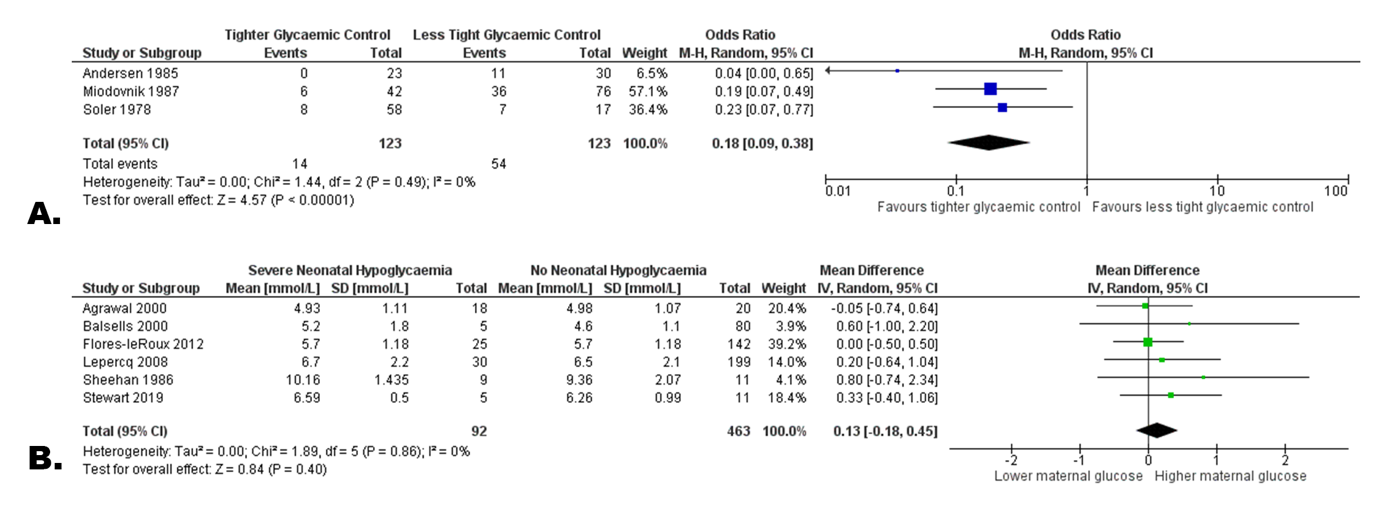
**Figure S1. Effect of tight compared to less tight or no intrapartum glycaemic control in women with diabetes on severity of hypoglycaemia.**

(A) Results from three cohort studies. (B) Results from six case control studies.
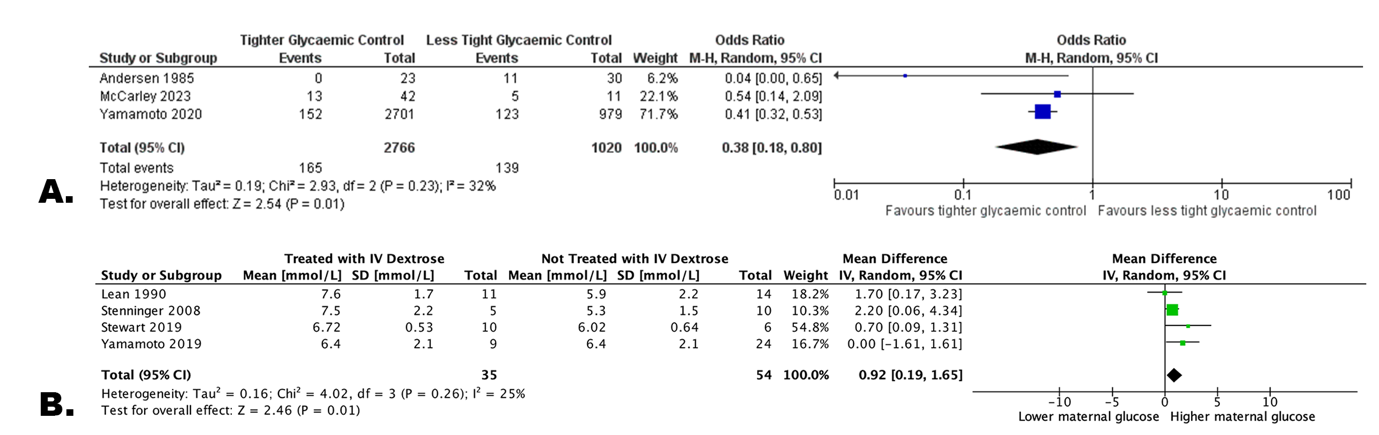


**Figure S2. Effect of tight compared to less tight or no intrapartum glycaemic control in women with diabetes on receipt of treatment for hypoglyacemia.**

(A) Results from three cohort studies. (B) Results from four case-control studies

**
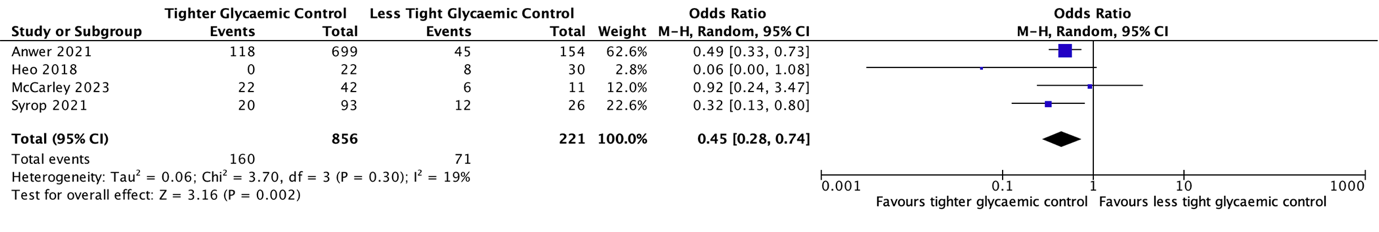
**

**Figure S3. Effect of tight compared to less tight or no intrapartum glycaemic control in women with diabetes on admission to special care nursery or neonatal intensive care nursery.**

Results from four cohort studies.

**
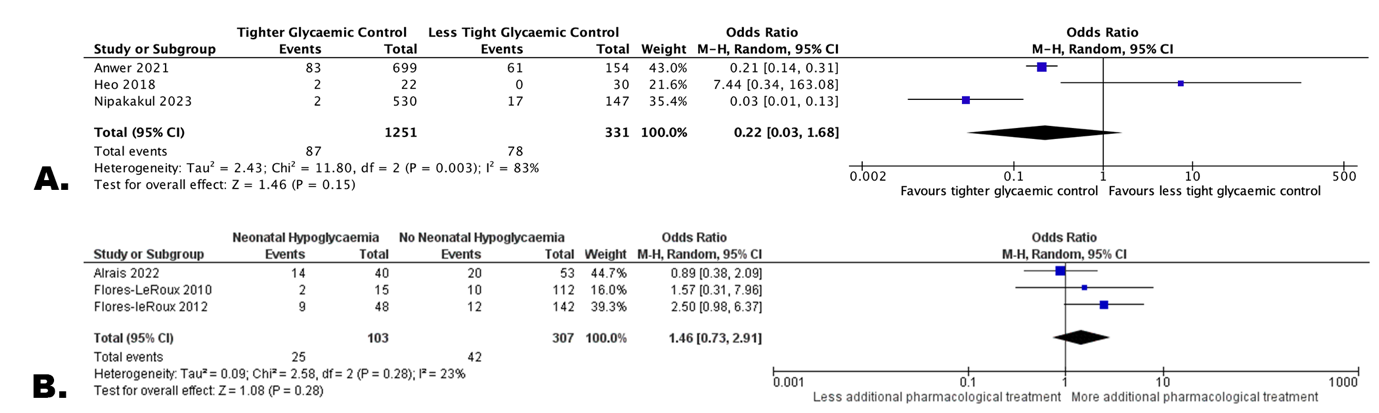
**

**Figure S4. Effect of tight compared to less tight or no intrapartum glycaemic control in women with diabetes on the use of additional intrapartum pharmacological treatment for maternal glycaemic control.**

(A)Results from three cohort studies. (B) results from three case-control studies


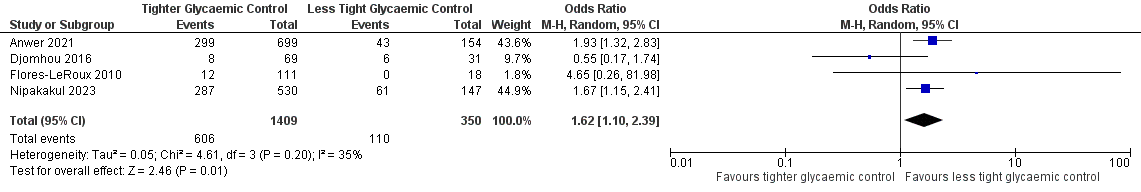


**Figure S5. Effect of tight compared to less tight or no intrapartum glycaemic control in women with diabetes on the mode of birth.**

Results from four cohort studies on Caesarean birth.
